# Application of Artificial Intelligence in Cardiology: A Bibliometric Analysis

**DOI:** 10.7759/cureus.66925

**Published:** 2024-08-15

**Authors:** Abubakar I Sidik, Roman N Komarov, Sidique Gawusu, Aliu Moomin, Malik K Al-Ariki, Marina Elias, Dmitriy Sobolev, Ivan G Karpenko, Grigorii Esion, Jonas Akambase, Vladislav V Dontsov, Abdul Majed I Mohammad Shafii, Derrar Ahlam, Naya W Arzouni

**Affiliations:** 1 Cardiothoracic and Vascular Surgery, RUDN University, Moscow, RUS; 2 Cardiothoracic Surgery, I. M. Sechenov University Hospital, Moscow, RUS; 3 Whiting School of Engineering, Johns Hopkins University, Baltimore, USA; 4 The Rowett Institute, University of Aberdeen, Aberdeen, GBR; 5 Cardiothoracic Surgery, RUDN University, Moscow, RUS; 6 Cardiology, European Medical Center, Moscow, RUS; 7 Cardiothoracic Surgery, A.A. Vishnevsky Hospital, Moscow, RUS; 8 Anatomy, Cairns Hospital, Cairns, AUS; 9 Cardiothoracic Surgery, Moscow Regional Research and Clinical Institute, Moscow, RUS; 10 Cardiovascular Medicine, RUDN University, Moscow, RUS

**Keywords:** machine learning, heart, deep learning, convolutional neural network, cardiology, cardiac, bibliometric analysis, artificial intelligence

## Abstract

Recent advancements in artificial intelligence (AI) applications in medicine have been significant over the past 30 years. To monitor current research developments, it is crucial to examine the latest trends in AI adoption across various medical fields. This bibliometric analysis focuses on AI applications in cardiology. Unlike existing literature reviews, this study specifically examines journal articles published in the last decade, sourced from both Scopus and Web of Science databases, to illustrate the recent trends in AI within cardiology. The bibliometric analysis involves a statistical and quantitative evaluation of the literature on AI application in cardiovascular medicine over a defined period. A comprehensive global literature review is conducted to identify key research areas, authors, and their interrelationships through published works. The leading institutions and most influential authors in research on the role of AI in cardiology were located in the United States, the United Kingdom, and China. This study also provides researchers with an overview of the evolution of research in AI and cardiology. The main contribution of this study is to highlight the prominent authors, countries, journals, institutions, keywords, and trends in the development of AI in cardiology.

## Introduction and background

Cardiovascular disease (CVD) refers to a class of diseases that is characterized by the malfunction or dysfunction of the heart and blood vessels, and it remains the leading cause of mortality and morbidity worldwide, according to WHO data [1]. While there has been considerable progress in medical treatments and interventions to address CVD, there is still much research to be done focusing on diagnostic approaches, clinical management, and preventive measures because of the highly diverse and intricate causative factors associated with CVD. Artificial intelligence (AI) has become one of the most significant technologies that has impacted several industries and has the capability of revolutionizing cardiology in the healthcare industry. AI includes machine learning (ML), deep learning (DL), and natural language processing (NLP), which can process copious input data, analyze data patterns, and deliver output data in the form of information understandable to humans [2].

AI is the ability of a computational system to replicate human intelligence in rational learning, decision-making, and control through the capability to comprehend language, apply comprehensibility, or analyze information and/or data with the application of various algorithms and cognitive computing mechanisms [3]. AI mainly uses ML, which employs models that are trained with data in order to make decisions and design solutions to particular problems [4]. DL is a form of learning algorithm within ML that comprises sophisticated features such as neural networks (NNs), acting intrinsically as a computer system that can recognize, create, and learn hierarchical data or information [5]. It entails the conversion of input data to what can be termed more complex output data. It is established that various cardiovascular disorders, including atherosclerosis, are associated with hereditary factors. The level of coronary artery calcium, which in many instances determines the possible severity of coronary atherosclerosis, can be predicted using advanced methods such as DL networks in conjunction with large-scale genome-wide association studies [6].

Diagnostic imaging of CVDs such as echocardiography, cardiac angiography, and ECG are real-time diagnostic tools based on DL. These diagnostic techniques are now routinely used to identify patients with heart conditions such as ischemic heart disease, arrhythmias, valve dysfunction, etc. Paroxysmal supraventricular tachycardia (PSVT) is a sudden, periodic, but repetitive arrhythmia that is associated with a worsening of quality of life in affected patients. PSVT, although treatable, has not been easy to diagnose because of its sudden and episodic nature, often occurring between normal sinus rhythms. Using modern deep ML-based ECG, it is now possible to capture episodes of discreet arrhythmias in order to diagnose PSVT at the initial stage [7].

Screening for congestive heart failure (CHF) is important to determine the prognosis and treatment methods to be assigned to CHF patients. NN models, which are relatively new in the field of cardiology, have been proven to be 85% accurate when applied in the diagnosis of CHF at the presymptomatic stage [7]. With the help of specific algorithms, AI can play a more significant role in the interpretation of the results of cardiac imaging methods such as echocardiography, cardiac CT, cardiac MRI, and EKG [7].

Rare complications from mitral valve repair or replacement include paravalvular regurgitation, which can often result in postoperative hemolytic anemia [8]. These complications are challenging to detect with cardiac imaging alone. Integrating AI into these imaging techniques can significantly enhance specialists’ ability to identify such issues before the development of symptoms. Some studies comparing mitral valve annuloplasty with different rings have reported no difference in short- and long-term clinical outcomes, despite echocardiography revealing differences in valve mechanics [9]. AI and ML can be instrumental in highlighting these contrasts.

A study performed in 2019 by Stanford University scientists in conjunction with Apple researchers reported that AI incorporated into smartwatches had the ability to successfully detect episodes of atrial fibrillation and atrial flutter. The technology analyzed pulse wave algorithms to almost accurately identify these cardiac arrhythmic episodes at the asymptomatic stage [10]. Presently, smartwatches are essential in diagnosing silent atrial arrhythmias, thus allowing early management. Cardiac MRI and CT scans of patients are now studied using AI and ML algorithms to diagnose different cardiac pathologies; this fastens the identification and therapy for these conditions, leading to a positive impact on patient outcomes. AI is trained with largely untapped three-dimensional imaging data for rapid analysis and interpretation of images in the future.

The application of AI in cardiology is a rapidly evolving field, demonstrating promising results in enhancing diagnostic accuracy, predicting patient outcomes, personalizing treatment plans, and improving overall patient care. AI-driven tools and algorithms are being integrated into various aspects of cardiovascular care, from imaging and electrophysiology to risk assessment and rehabilitation [11].

As digital health usage and personal data collection grow, it is essential to address the associated ethical principles and privacy risks. Ensuring data integrity and integrating legal and ethical guidelines are necessary to mitigate the potential risks of digitization [12]. Using data responsibly is crucial to safeguarding individual liberty, privacy, and autonomy. Although high-quality data is essential for effective AI development, it must be obtained and used ethically. Medical AI algorithms need to be trained using carefully selected patient data to ensure their accuracy and reliability for individual patients. However, we must consider how to safeguard sensitive patient data, especially as it becomes more valuable and the boundary between innovation and exploitation blurs.

The source of the training dataset could lead to AI-associated bias and discrimination. For instance, disease-detection algorithms trained predominantly on data from Caucasian populations may yield less accurate or potentially incorrect diagnoses for other populations if the training and validation data lack diversity [13]. Therefore, without coordinated efforts to enhance inclusiveness by training on datasets that are representative of various races, ethnicities, genders, and socioeconomic statuses, ML and AI technologies could potentially worsen existing inequities in healthcare [14].

As research on AI’s role in cardiology grows, it is increasingly important to analyze trends, patterns, and the impact of these technological advancements. Doing this will guide future research directions and the clinical applications of the new technology. This bibliometric analysis is centered on AI applications, specifically in the field of cardiology. Unlike previous literature reviews published on this subject, this review was limited to papers that were published in the last 10 calendar years. The included articles, which were sourced from the Scopus and Web of Science databases, depict the current trends in the integration of AI in the management of CVDs.

By systematically identifying research trends, gaps, and directions that have not been fully explored, this methodology provides a comprehensive overview that the existing body of work lacks. The purpose of this paper is to review research on the application of AI in cardiology over the last decade using the science mapping review methodology. The review addresses the following research questions (RQ): RQ1: What are the publication trends and growth patterns in artificial intelligence research related to cardiology over time? RQ2: What authors, countries, institutions, journals, and articles have had the greatest influence on the use of artificial intelligence in cardiology research over the past decade? RQ3: What is the intellectual structure of the knowledge base on artificial intelligence in cardiology? RQ4: What topics in the artificial intelligence literature have been studied with the greatest frequency and are currently attracting the greatest attention?

By addressing these questions, this bibliometric analysis aims to provide a comprehensive overview of the current state of research on AI in cardiology, highlight significant contributions, and identify gaps and opportunities for future research. It will provide a comprehensive analysis of the available literature on the use of AI in the field of cardiology [15-18]. It will also serve as a valuable resource for researchers, clinicians, and policymakers seeking to understand and leverage AI’s potential to improve cardiovascular health outcomes. This is the first effort aimed at “science mapping” the full literature in this domain of the application of AI.

With the help of bibliometric methods, 1,791 documents sourced from the Scopus and Web of Science databases were analyzed. Descriptive statistics were used to establish patterns that identified the developmental trends and content of the literature on AI’s application in cardiology. Additionally, citation, co-citation, and keyword frequency co-occurrence analyses were performed for the evaluation of the trends in authorship, document types, and topics.

## Review

Methodology

Data Collection and Description

**Figure 1:**
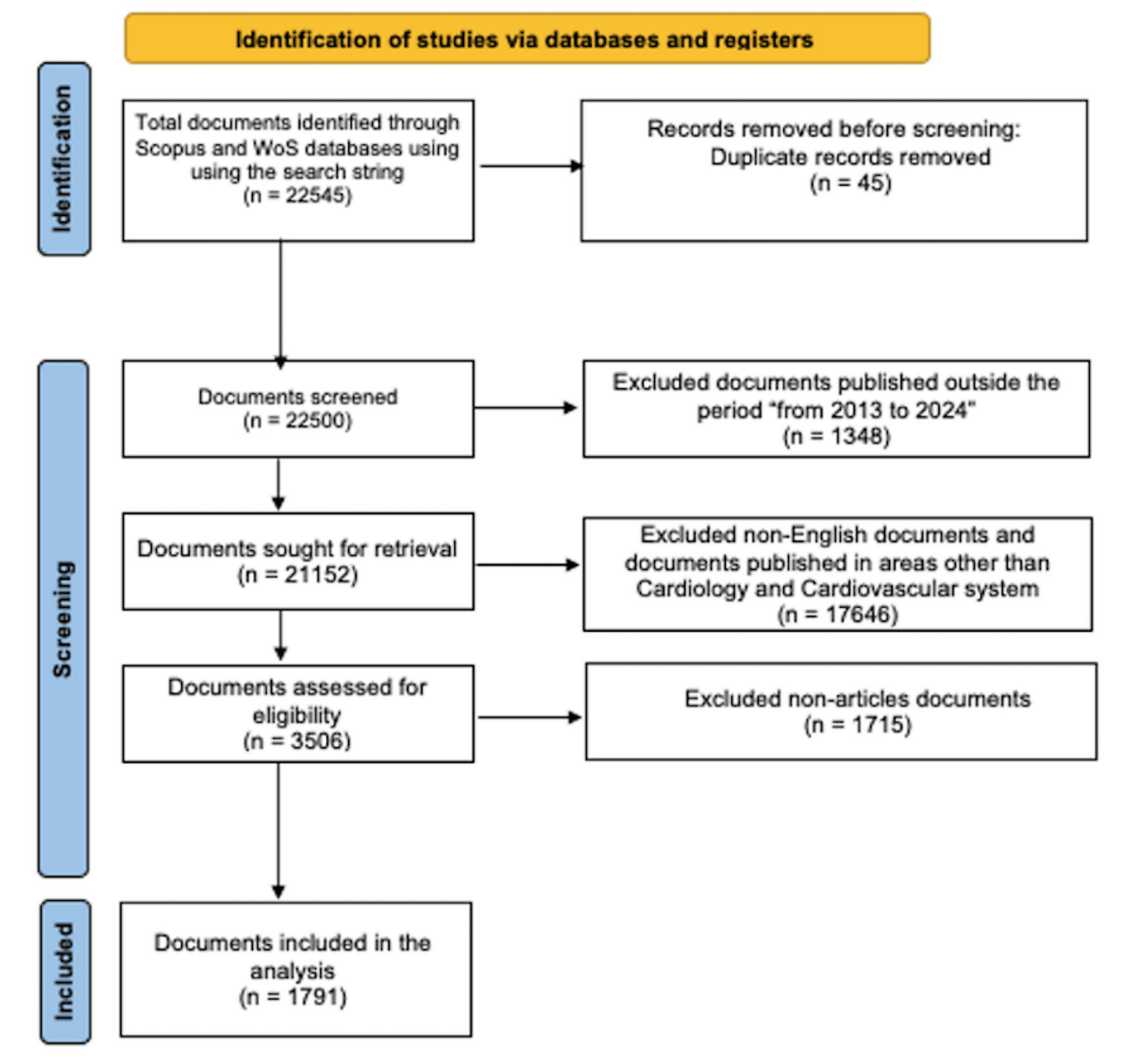
PRISMA flow diagram Flow diagram detailing steps in the identification and screening of sources. PRISMA, Preferred Reporting Items for Systematic Reviews and Meta-Analyses Adapted from Moher et al. (2009) [19]

The initial sample was obtained through research in the Scopus and Web of Science databases, followed by filtering and technical data treatment (Figure 1). The choice of these bases was made considering the relevance of indexed articles published in journals with a high impact factor (Journal Citation Report and CiteScore), as well as the ease of stratification and access to publications. Furthermore, the Medline database, which constitutes the primary component of PubMed, is 100% included within Scopus, and therefore, using Scopus will automatically include publications in Medline as well.

To retrieve the bibliographic records, this search string was used in the databases: [("artificial intelligence" OR "AI" OR "machine learning" OR "deep learning" OR "convolutional neural network") AND ("cardi*" OR "heart")]. The inclusion criteria were set to select only the peer-reviewed articles published in English from January 2013 to June 2024, focusing on studies about the application of AI in cardiology and cardiovascular systems.

Following the initial data retrieval, a meticulous data cleaning process was undertaken. This involved the removal of duplicate entries resulting from the overlap between the databases and the refinement of search strings to weed out irrelevant articles. The relevance of each article was assessed based on its title, abstract, and keywords, ensuring a focus on the study’s primary objective. The iterative optimization of search strings was instrumental in refining the dataset into a manageable and relevant collection of literature for analysis. The search resulted in 22,545 publication articles, out of which 1,791 were selected for bibliometric analysis using Preferred Reporting Items for Systematic Reviews and Meta-Analyses (PRISMA) [18,19]. This selection process is depicted in Figure 1.

Data Analysis

Publication information such as the title, authors’ names, authors’ institutions, and citations that were obtained from the 1,791 documents were saved for analysis. The data analysis involved the use of descriptive statistics and bibliometric methods such as citation, author co-citation, and author keyword co-occurrence analysis; this was achieved with the help of Scopus and Web of Science analytical tools, in addition to Microsoft Excel, Bibliometrix, and VOSviewer [20].

Key metrics were derived to highlight trends in publication volume over time, identifying the most influential articles and authors through citation analysis. The study also mapped out co-authorship networks to reveal patterns of collaboration among researchers and institutions, providing insights into the community’s structure. Keyword analysis further allowed the examination of research foci, emerging trends, and potential gaps in the existing literature, highlighting areas ripe for future exploration.

Results

Descriptive Trends in the Literature

**Figure 2:**
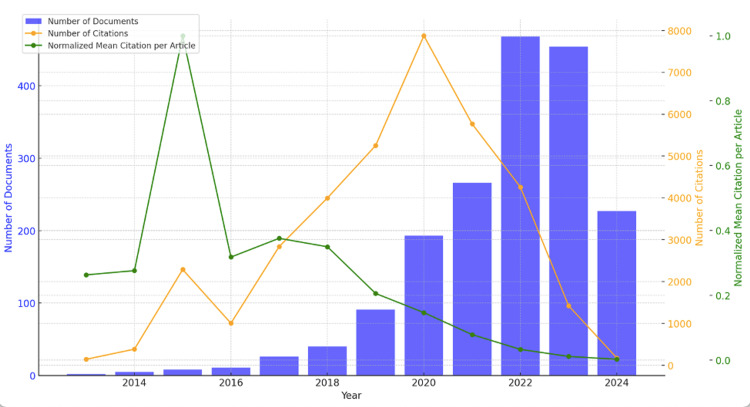
Annual publications, citations, and normalized citation impact This graph depicts the longitudinal evolution of the literature on the role of AI in cardiology (2013-2024) in terms of annual publications, citations, and normalized citation impact (n = 1,791). AI, artificial intelligence

Publications sourced for this review were published between January 2013 and June 2024. Scholarly interest in this field (blue bars) increased gradually, reaching a peak of 468 publications in 2022 before declining slightly in 2023. So far, the number of publications for the year 2024 (January to June) is 227. The trend in the yearly output of documents shows that interest in the application of AI in cardiovascular medicine is continuously increasing. The number of citations (orange line) rose steadily over the years to reach a peak of 7,873 citations in 2020; afterward, it declined sharply. This can be explained by the fact that papers published during the nascent stage of research on the application of AI in cardiovascular medicine were the main sources for articles published later on; moreover, the earlier publications have been in circulation much longer than newer publications. The normalized mean citation per article (green line) adjusts the average citations per article relative to the highest value observed in 2015. This normalized metric shows a peak in 2015 and a continuous decline in recent years, indicating that despite the increasing number of publications, the average impact of each article has decreased (Figure 2).

Analysis of the Influential and Active Authors, Countries, Institutions, Journals, and Articles on the Use of AI in Cardiology

**Figure 3:**
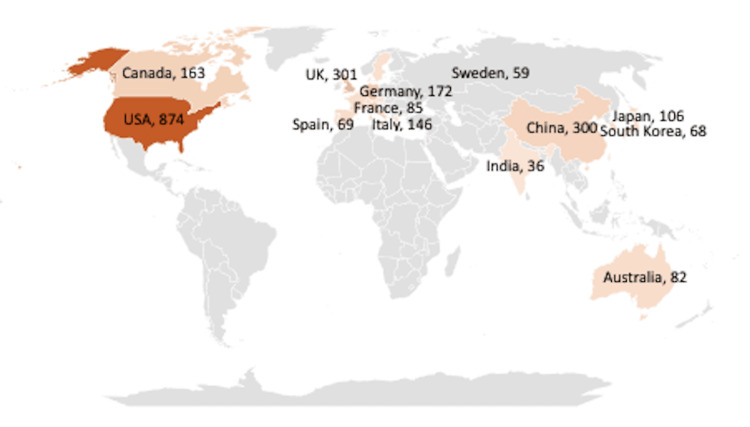
Global distribution of publications of AI in cardiology This figure represents the worldwide distribution of the literature on the application of AI in cardiology in 2013-2024 (n = 1,791). AI, artificial intelligence

The countries of origin of the authors were analyzed to determine the regions most interested in research on the application of AI in cardiology. The reviewed literature was developed in 82 different countries around the globe, thus demonstrating the international nature of interest in the subject (Figure 3). Nonetheless, half of the scholarly work was produced by scholars in the United States (48.8%), followed by the United Kingdom (16.8%), China (16.8%), Germany (9.6%), and Canada (9.1%). These four countries are the sources of most of the existing literature on this topic in this review.

**Figure 4:**
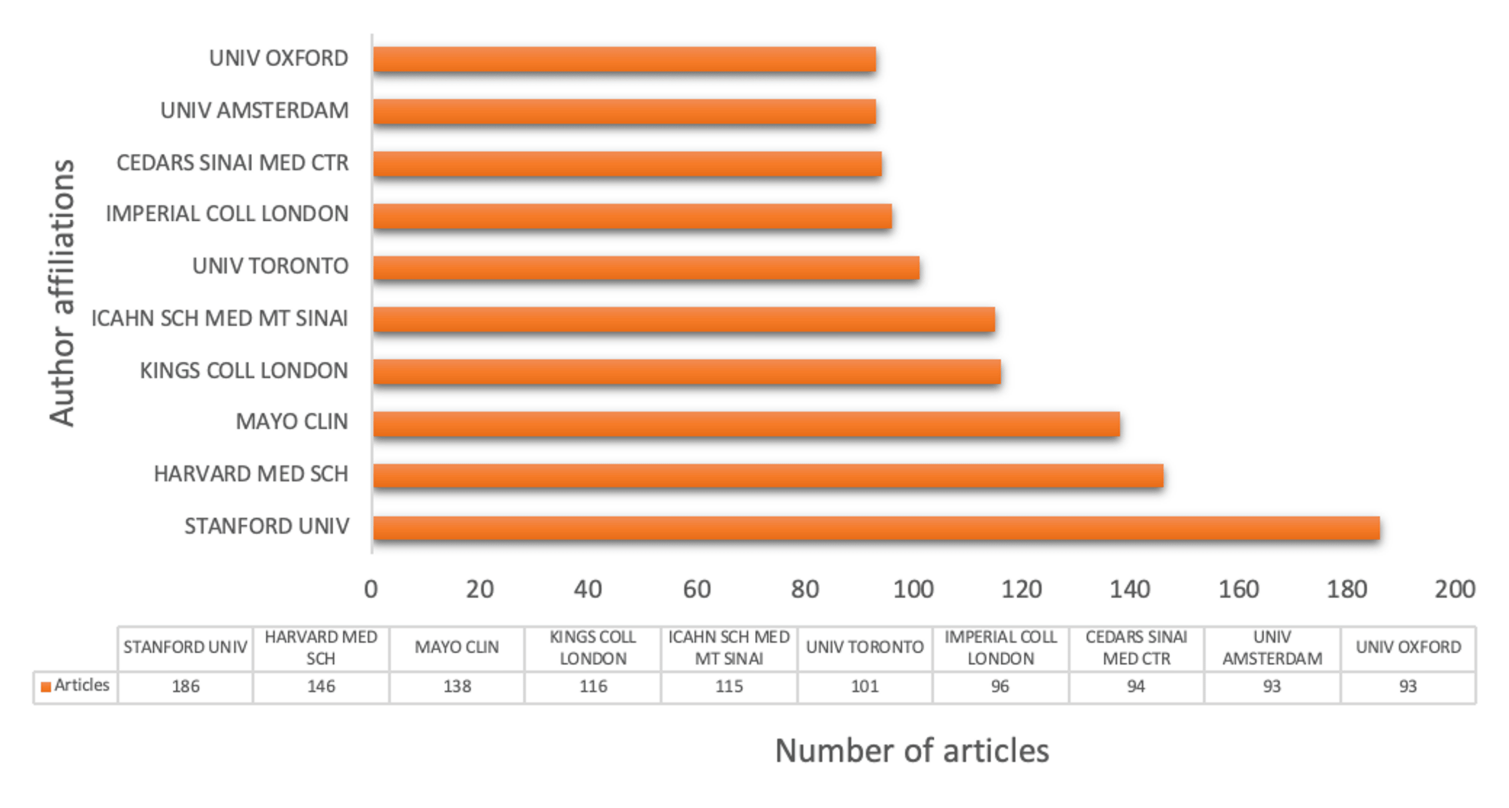
Number of publications by institutions This figure demonstrates the most active author affiliations in research on the use of AI in cardiology. AI, artificial intelligence

An analysis of the authors’ affiliations revealed a concentration of work on the implementation of AI in cardiology in Western institutions (Figure 4). Universities in the United States and the United Kingdom dominate research on AI in cardiology. This is associated with the fact that reputable institutions such as Stanford University, Harvard Medical School, Mayo Clinic, and Imperial London College possess state-of-the-art research facilities and are well-funded, giving them an advantage in newer research areas like the incorporation of ML into healthcare.

Table 1 shows the result of the Scopus citation analysis of the 15 most cited authors. Our database review revealed 13,151 scholars listed as authors or co-authors, which depicts a widespread interest in this research area among the scholarly community. However, it should be noted that the majority of the published papers were from researchers in the United States, the United Kingdom, and China; authors from these countries published a substantial number of articles on the role of AI in cardiology (Figure 1). For instance, the most productive scholar is Slomka PJ from the United States, who published 37 papers. These findings demonstrate a growing area of study among scientists from some of the world’s largest economies who have strong interests and perspectives on the benefits of AI to cardiology.

**Table 1:** Rank order of the most influential authors on the application of AI in cardiology by Scopus citations and documents published AI, artificial intelligence

Authors	Nation	Articles	Total citations	Citations per document	h-index
Deo RC	United States	7	2,218	316.8	33
Slomka PJ	United States	37	1,988	53.7	13
Sengupta PP	United States	23	1,637	71.2	6
Dey D	United States	33	1,534	46.5	55
Berman DS	United States	27	1,305	48.3	9
Shameer K	United States	5	1,171	234.2	9
Rueckert D	United Kingdom	11	1,147	104.3	97
Dudley JT	United States	5	1,091	218.2	13
Johnson KW	United States	6	1,073	178.8	25
Bai W	United Kingdom	7	1,071	153	37
Petersen SE	United Kingdom	21	942	44.9	15
Friedman PA	United States	22	921	41.9	1
Glicksberg BS	United States	5	906	181.2	37
Noseworthy PA	United States	24	897	37.4	11
Attia ZI	United States	20	813	40.7	61

The data presented in Table 1 indicate that the most influential authors in the literature on AI in cardiology have been Deo RC (2,218 Scopus citations), Slomka PJ (1,988), Sengupta PP (1,637), Dey D (1,534), and Berman DS (1,305). We note that the citation impact of the authors is very high. To clarify, Table 1 reflects citations solely from documents included in our review database for each author. The Scopus h-index associated with each author encompasses their entire body of scholarly work, which extends beyond AI in cardiology. Furthermore, our selection of the top 15 influential authors for this analysis means that several other active authors who have authored numerous, albeit less impactful, documents are not included in the table.

Table 2 presents the most influential publications in the realm of AI applications in cardiology, based on total citations in Scopus. Three of these documents have accrued over 500 citations each [21-30]. Given the relative newness of AI literature in medicine, these citation numbers are substantial. It is important to note that Scopus citations typically appear lower compared to Google Scholar due to its more selective coverage of literature [31], although both metrics generally show a strong correlation. Furthermore, the data in Table 2 underscores the significant impact that research reviews have contributed to advancing this field of study.

**Table 2:** Most influential documents on the application of AI in cardiology by Scopus citations, 2013-2024 (n = 1,791) AI, artificial intelligence; NTC, normalized total citations; TC, total citations

Rank	Author	Article	Journal	TC	NTC
1	Deo (2015) [21]	Machine learning in medicine	Circulation	1,590	6.06
2	Kornej et al. (2020) [22]	Epidemiology of atrial fibrillation in the 21st century: novel methods and new insights	Circulation Research	551	14.31
3	Johnson et al. (2018) [23]	Artificial intelligence in cardiology	Journal of the American College of Cardiology	506	5.52
4	Krittanawong et al. (2017) [24]	Artificial intelligence in precision cardiovascular medicine	Journal of the American College of Cardiology	474	4.81
5	Zhang et al. (2018) [25]	Fully automated echocardiogram interpretation in clinical practice: feasibility and diagnostic accuracy	Circulation	454	4.95
6	Motwani et al. (2017) [26]	Machine learning for prediction of all-cause mortality in patients with suspected coronary artery disease: a 5-year multicentre prospective registry analysis	European Heart Journal	454	4.61
7	Bai et al. (2018) [27]	Automated cardiovascular magnetic resonance image analysis with fully convolutional networks	Journal of Cardiovascular Magnetic Resonance	406	4.43
8	Chen et al. (2020) [28]	Deep learning for cardiac image segmentation: a review	Frontiers in Cardiovascular Medicine	398	10.34
9	Ambale-Venkatesh et al. (2017) [29]	Cardiovascular event prediction by machine learning: the multi-ethnic study of atherosclerosis	Circulation Research	337	3.42
10	Goldstein et al. (2017) [30]	Moving beyond regression techniques in cardiovascular risk prediction: applying machine learning to address analytic challenges	European Heart Journal	307	3.12

In terms of the foci of AI application in cardiology, the highly cited documents were concentrated in four journals. The most highly cited document, Deo et al. [21], was a review article that centered on identifying problems in medicine that might benefit from ML-based large medical data sets and algorithms. The two main challenges the authors identified were that translating ML algorithms into clinical practice has been slow, the need for domain-specific expertise, the complexity of medical data, and the conservative nature of clinical practice. The authors made the following conclusions: while ML holds significant promise for advancing medical practice, overcoming existing barriers is essential. Future efforts should focus on amassing diverse training data, leveraging domain-specific knowledge, and fostering collaborative environments to drive innovation in healthcare.

Kornej et al. [22] reviewed research on the role of AI in the prevention and management of atrial fibrillation. They explored trends in diseases, opportunities for disease prevention related to underlying risk factors and concurrent conditions, current advancements in diagnostics and predicting risks, and the prognostic implications of atrial fibrillation and its complications. Lastly, they examined recent technological developments, such as eHealth, and methodological advancements in AI, assessing their importance for future prevention and management of diseases. The authors concluded that AI, particularly through supervised and unsupervised learning methods, plays a significant role in the prevention and management of atrial fibrillation. By improving risk stratification, enhancing predictive models, automating ECG interpretation, facilitating personalized treatment, and enabling continuous monitoring, AI contributes to better patient outcomes and more efficient management of this common cardiac arrhythmia.

Johnson et al. [23] conducted a research review on “Artificial intelligence in cardiology,” offering clinicians a comprehensive overview of AI and ML relevance to the field. The review examines current applications of these technologies in cardiology and predicts future integrations of AI into cardiology practice. Initially, the review explores predictive modeling concepts that are essential in cardiology, including feature selection and common drawbacks such as improper categorization. Secondly, it discusses popular algorithms used in supervised learning and highlights specific AI applications in cardiology and related disciplines. Thirdly, it discusses the emergence of DL and similar methods, together referred to as unsupervised learning; it also provides examples relevant to the application of unsupervised learning in general and cardiovascular medicine. The paper emphasizes the potential of these technologies to enhance precision in cardiology and improve patient outcomes. Ultimately, it asserts that AI integration in cardiology should be embraced by clinicians as it promises enhanced patient care through deeper data interpretation capabilities.

The next two most cited papers [24,25] respectively examined AI’s role in diagnosing and predicting CVDs and detailed the training and assessment of convolutional NN models that are employed in a multitude of tasks, notably in diagnosing cardiac conditions through echocardiographic imaging. Krittanawong et al.’s [24] review paper gave a glimpse of AI’s application in cardiovascular clinical care and discussed its potential role in facilitating precision cardiology. The research by Zhang et al. [25] described how they developed models to detect hypertrophic cardiomyopathy, cardiac amyloid, and pulmonary arterial hypertension, which serves as the groundwork for using automated interpretation to support serial patient tracking and scalable analysis of millions of echocardiograms archived within healthcare systems.

Collaboration Networks and International Partnerships in AI Research and Intellectual Structure of the Knowledge Base of Application of AI in Cardiology

Researchers who use science mapping methods to review literature have also investigated the “intellectual structure” of different fields of study [16]. This structure refers to the main theoretical and empirical lines of inquiry, or “schools of thought,” that define a particular field of research. In our study, we used author co-authorship and co-citation analyses in VOSviewer to create network maps. These maps offer insights into the intellectual framework of the application of AI in cardiology.

**Figure 5:**
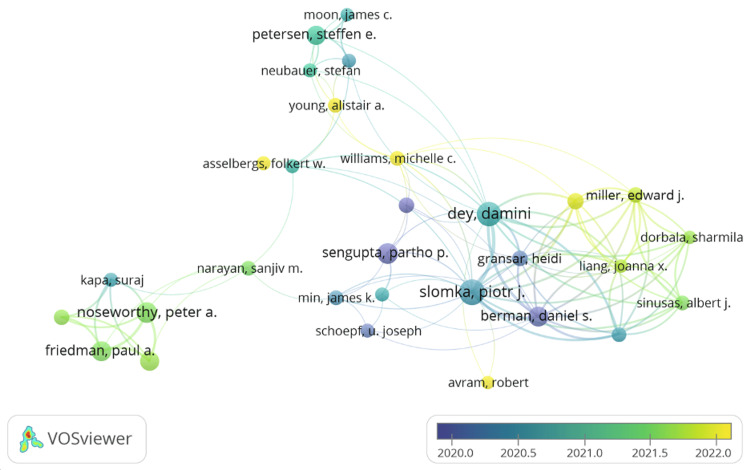
Author co-authorship analysis map This figure depicts author collaboration in research on the application of AI in cardiology (threshold of 25 co-authors per document, minimum of 10 documents, and 10 citations per author, selected 30 most related authors, five clusters). AI, artificial intelligence

VOSviewer was set to a threshold of at least 10 documents by an author and a minimum of 10 citations of an author, which yielded 37 scholars; however, the co-authorship map displays the 30 most connected scholars (Figure 5). The central hub of authors (blue-green cluster) including Dey D (United States), Slomka PJ (United States), and Berman DS (United States) indicates a core group with significant influence and high-level collaboration. These authors have co-authored multiple publications on the application of AI, ML, and DL in the diagnosis of coronary artery disease using coronary artery calcification quantification, coronary calcium score, and myocardial perfusion imaging [32-36]. The peripheral clusters with authors such as Petersen SE (United Kingdom), Neubauer S (United Kingdom), Moon JC, and Young AA (United Kingdom) suggest fewer direct collaborations or that they are part of smaller, perhaps specialized, collaborative groups. Petersen SE and Neubauer S have co-authored five papers that focused on research on the application of AI and ML in cardiac MRI [37-41].

The green-yellow clusters, consisting of authors such as Dorbala S (United Kingdom), Sinuses AJ (United States), and Miller EJ (United States), have entered the collaborative network on the adoption of AI in cardiology more recently. These authors have worked together on eight papers centered on the role of AI and ML in single-photon emission CT myocardial perfusion imaging [42-49]. The other peripheral green cluster comprises Noseworthy PA (United States) and Friedman PA (United States), who have together authored published papers on AI-based electrocardiography models for detecting hypertrophic cardiomyopathy, heart failure, atrial fibrillation, and left atrial myopathy [32,34,50-52].

The collaboration mapping reflects the most influential and active countries in research on this topic. The map also shows that there was a strong collaboration among scholars from the United States and the United Kingdom in the early, middle, and later parts of the last decade. Analysis of the work they collaborated on indicates diagnostic cardiology is their main area of interest.

The co-citation analysis assessed how often pairs of authors were referenced together in the reference lists of the 1,791 documents included in our review database. Unlike Scopus citation analysis, which focuses on citations of specific documents, co-citation analysis examines a broader body of literature (the references cited by authors within the reviewed documents). Scholars who employ co-citation analysis suggest that authors frequently co-cited by others likely share similar research perspectives. Using VOSviewer software, we generated a network map based on the frequency of “author co-citations,” which visually represents similarities among the authors referenced in our literature database [20].

**Figure 6:**
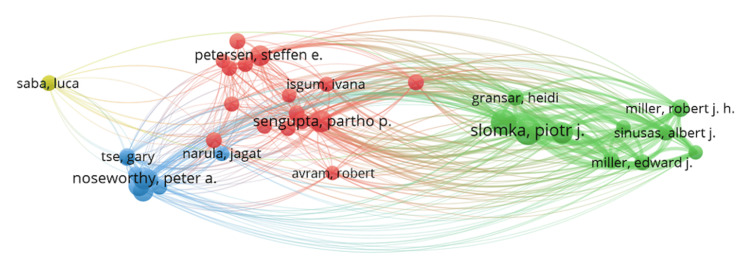
Author co-citation analysis map of the literature on the role of AI in cardiology This figure illustrates how authors of literature on AI in cardiology are co-cited (threshold: 25 co-authors per document, a minimum of 10 documents and 10 citations per author, selected 37 authors, four clusters). AI, artificial intelligence

VOSviewer was configured with criteria requiring each author to have published at least 10 documents and cited at least 10 times, resulting in a display of 37 scholars on the co-citation map (Figure 6). In this visualization, larger bubbles represent scholars who are more frequently co-cited, indicating their influence in the field. The map uses colored clusters to categorize scholars into groups based on their co-citation relationships, illustrating distinct schools of thought within the research. The author’s co-citation map shows that the intellectual structure of AI in cardiology literature is comprised of four schools of thought. Key authors like Slomka PJ, Sengupta PP, and Noseworthy PA in the center of the clusters have the highest density of connections, indicating strong and frequent collaborations. These authors in the central network of each cluster form the backbone of the research collaboration. They are pivotal in driving research within this domain.

The major prominent co-cited authors in the green cluster are Slomka PJ, Miller EJ, and Gransar H, who have worked on the use of AI tools in the analysis of cardiac diagnostic results to predict morbidity and mortality risk [33,36,53]. Sengupta PP, Asselbergs FW, and Petersen SE are some of the most influential authors in the red cluster. They have each done research on the adoption of AI and ML in interpreting echocardiographic, cardiac MRI, and cardiac CT results [54-56]. The main influential authors co-cited in the blue cluster, Noseworthy PA and Tse G, have researched the application of AI and ML in the diagnosis of cardiac arrhythmia [57,58]. The peripheral clusters with authors like Noseworthy PA, Friedman PA, Miller RJ, and Sinusas AJ represent authors detached from the most influential authors with more specialized or independent collaboration networks.

Topical Foci of the Role of AI in Cardiology Knowledge Base

The authors’ keyword co-occurrence analysis was used to analyze the topics studied in the literature on the role of AI in cardiology. We first used VOSviewer to identify the most frequent keywords. The minimum number of occurrences of a word was set at 10, and 3,075 author keywords were found. Merging synonyms resulted in 3,021 keywords, 80 of which met the threshold. The 10 most frequently occurring keywords were machine learning (725), artificial intelligence (480), deep learning (271), heart failure (255), coronary artery disease (160), echocardiogram (142), cardiac MRI (127), arrhythmia (123), electrocardiogram (111), and cardiac CT (79).

**Figure 7:**
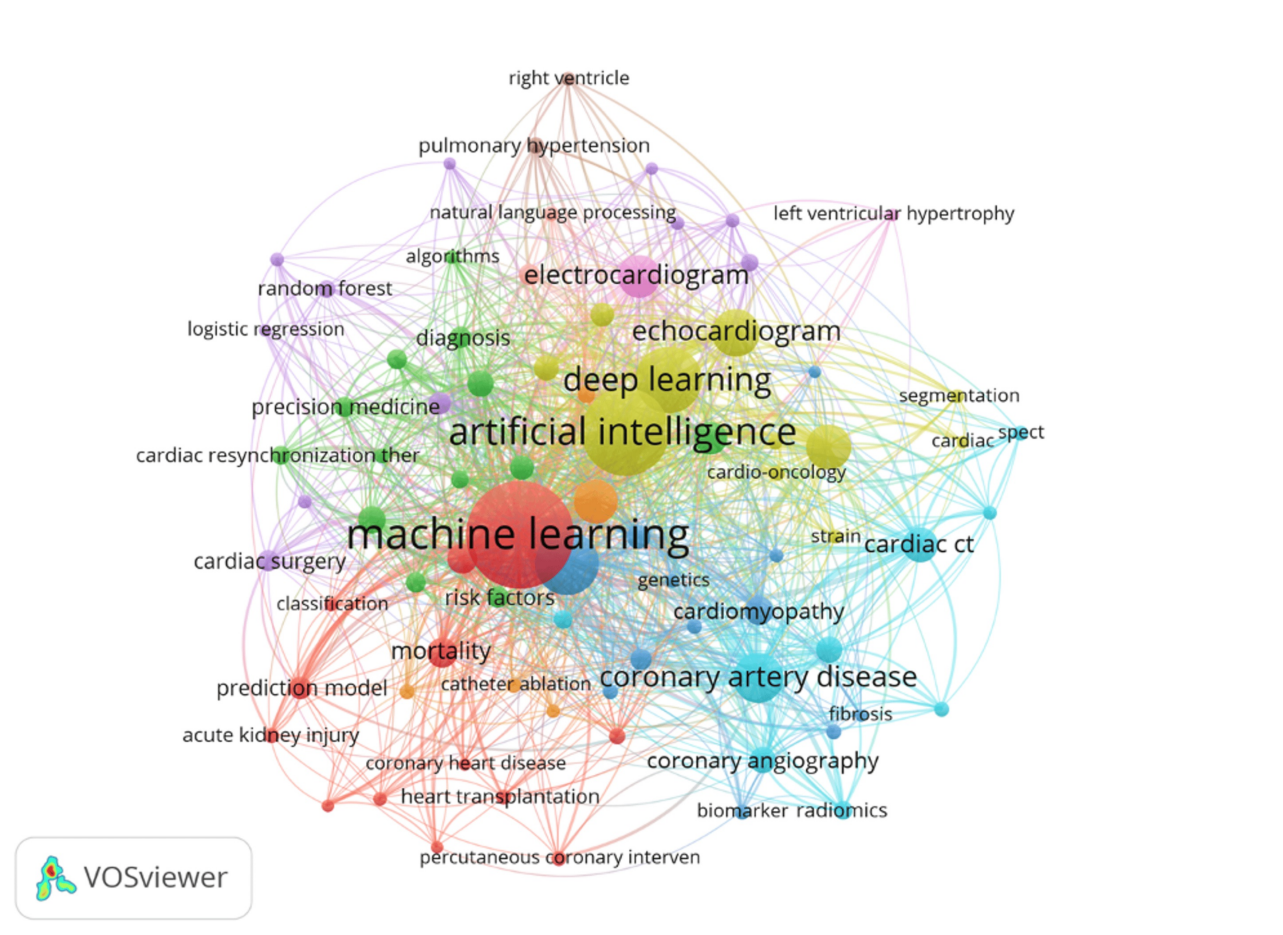
Keyword network visualization map This figure is a representation of authors’ keywords co-occurrence in the literature on AI in cardiology, January 2013-June 2024 (threshold: 10 occurrences, display 80 keywords). AI, artificial intelligence

As indicated in the network visualization map (Figure 7), prominent keywords like “machine learning” and “artificial learning” illustrated with the largest and most central nodes represent the core methodologies and focal points in this research area. “Deep learning” and “electrocardiogram” are also prominent, suggesting that DL techniques are particularly relevant to the analysis and interpretation of ECG data. The author’s keyword co-citation map in Figure 7 shows that the intellectual structure of the literature on the role of AI in cardiology is comprised of two key themes. The first theme is Medical and Diagnostic Applications: keywords like “coronary artery disease,” “echocardiogram,” “cardiac CT,” “cardiac MRI,” and “biomarker radiomics” indicate a strong focus on applying ML and AI to medical imaging and diagnostics. The other theme is CVDs and Interventions: terms such as “coronary artery disease,” “coronary angiography,” “heart transplantation,” and “percutaneous coronary intervention” show a focus on cardiovascular health and related medical procedures. The visualization map also indicates that NLP, precision medicine, and genomics are emerging and specialized topics that suggest a trend toward personalized healthcare approaches.

**Figure 8:**
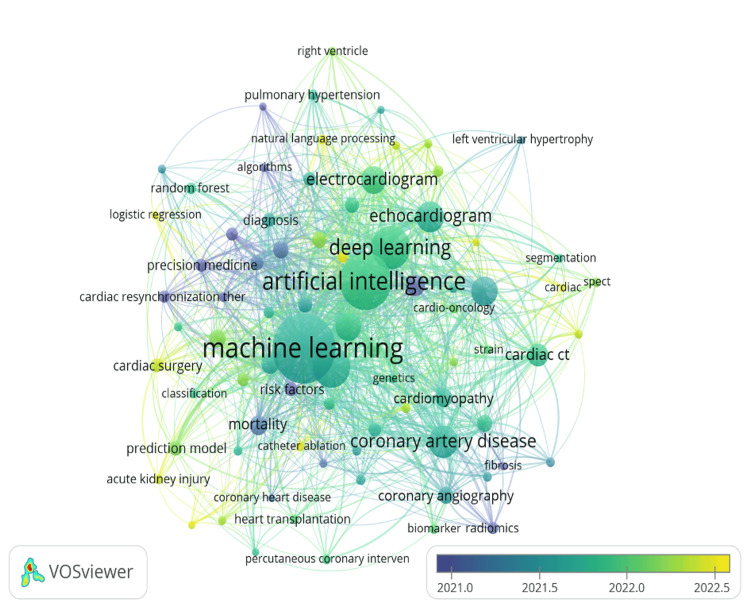
Temporal co-word map of the literature on AI in cardiology This figure shows the authors’ keywords evolution over time based on the publication dates of the documents. AI, artificial intelligence

Subsequently, a “temporal co-word map” (Figure 8) was created using VOSviewer by applying a threshold of 10 or more keywords co-occurrence [20]. This analysis examines how keywords have evolved over time based on the publication dates of the documents. Bubbles shaded in yellow or lighter colors represent topics that have recently gained scholarly interest in the field, while darker bubbles indicate topics that were more prominent in earlier periods. The interpretation of this map focuses on the size of each bubble (indicating frequency), its color (reflecting recency), and its position relative to other bubbles (indicating relationships between topics).

ML and AI are the most prominent themes, indicating they are central to the research topics analyzed. These two keywords have the most links (1,745 and 1,261, respectively) to other topics and are of current interest. Risk factors, mortality, and diagnosis are also prominent, highlighting the focus on identifying and predicting cardiovascular health outcomes. These findings reprise the discussion of the intellectual structure of the knowledge base, in which all the schools of thought (13 clusters) had a strong focus on the application of ML and AI in all aspects of cardiology.

Discussion

This review of research revealed the wide size of the literature on the application of AI in cardiology. We limited our research to documents published starting from 2013 because most of the literature on this topic was published in the past 10 years as a result of the gradual rise in interest in this field within this time frame. This can also be associated with the increasing recognition of the relevance of AI in disease risk stratification, diagnosis, therapy, and prevention.

The contribution of this study lies in its systematic synthesis of the literature, which has mapped the intricate web of research activities and collaborations. By doing so, it has outlined the current state of knowledge and provided a scaffold upon which upcoming studies can be built. This work serves as a crucial touchstone for researchers, clinicians, and policymakers alike, guiding their efforts in advancing the adoption of AI and ML in cardiology. Sustaining and expanding research in this area is not just scientifically pertinent; it holds the promise for tangible improvements in patient outcomes and overall public health.

A significant advancement in cardiovascular medicine has been the integration of AI into diagnostic tools, outcome predictions, and heart failure (HF) management. Using DL components of AI, especially artificial and convolutional NN, for HF diagnosis, combined with remote monitoring of at-risk patients through eHealth, can significantly decrease mortality related to structural heart diseases, particularly HF. Although AI has the potential to transform medical diagnosis, treatment, clinical care, risk prediction, and drug discovery by efficiently interpreting large databases beyond the capacity of the human brain, it faces limitations as a result of the lack of a supportive healthcare system and a shortage of trained clinicians capable of integrating AI models into their clinical decision-making and patient monitoring. Therefore, creating a strong link between AI models and clinical practice is crucial. [59]. Quality improvement studies often recommend periodic audits to monitor the impact of the implementation of action plans [60]; AI can become an essential tool to electronically evaluate improvements in the adoption of action plans in a more efficient manner.

Our analysis of the global geographic distribution of literature on the use of AI in cardiology confirmed that interest in this domain is global in scope. At the same time, however, we noted that knowledge on this matter has been predominantly generated by scholars and institutions in the United States, the United Kingdom, China, Germany, and Canada, with a paucity of contributions from other developed countries. This unbalanced distribution of research suggests a gap in this literature given the importance of AI in healthcare and the role it could play in identifying and giving a logistical understanding of endemics and pandemics that usually start in underdeveloped locations. We therefore urge scholars as well as funding agencies to also prioritize research on the significance of AI in the medical field in developing countries.

The collaboration networks reveal a high degree of international cooperation, which is crucial for the development and dissemination of AI technologies in medicine. Such collaborations not only facilitate the sharing of knowledge and resources but also help in addressing complex cardiovascular challenges through a multidisciplinary approach. Citation analysis identified the 15 most influential authors researching this issue. They are affiliated with two developed countries, namely the United States (12 authors) and the United Kingdom (three authors). These highly cited scholars have focused on cardiology in healthcare policy, healthcare innovation, healthcare management, and healthcare delivery.

The most influential studies, as identified by citation analysis, serve as cornerstones for subsequent research, often providing the empirical foundation that shapes future study designs, hypotheses, and the exploration of novel therapeutics. These pivotal works, such as those prominent within citation networks, have significantly contributed to the field by elucidating how AI can be incorporated into healthcare in order to optimize patient care. The analysis of highly cited papers indicates that the most impactful research focuses on the use of ML and DL techniques in areas such as cardiac imaging, risk assessment, and personalized treatment strategies. These advancements underscore the potential of AI to transform cardiovascular care by enhancing diagnostic accuracy, predicting patient outcomes, and optimizing therapeutic interventions.

The network visualization map of authors’ keywords co-occurrence provides a comprehensive overview of the thematic structure and interconnections within a research domain focused on AI and ML applications in medical and cardiovascular contexts. The centrality of AI and ML highlights their importance, while the diverse keywords around them show the wide range of applications and methodological approaches being explored. Emerging trends such as NLP and precision medicine suggest areas of growing interest and future research potential. This visualization helps researchers identify key themes, understand the interconnectedness of various topics, and track the evolution of research trends.

The bibliometric map of authors’ keywords temporal co-occurrence highlights the significant role of AI and ML in recent cardiovascular research, with a strong emphasis on diagnostics and predictive analytics. This study’s findings underscore the critical focus areas, such as the adoption of ML and AI in the management of heart failure, coronary artery disease, and arrhythmia, and the application of ML and AI in cardiac diagnostic imaging such as echocardiogram, MRI, ECG, and CT. Emerging trends like precision medicine and NLP are gaining traction, reflecting the evolving landscape of medical research toward integrating advanced technologies. The visualization provides a comprehensive overview of the research landscape, showing both established and emerging areas in the field.

Limitations

One limitation of this study stems from its reliance on quantitative methods for reviewing documents on the application of AI in cardiology. The review primarily analyzed bibliographic data associated with these documents rather than directly examining research findings. As a result, the study provides broad insights into the field’s overall development rather than synthesizing specific study outcomes. Another limitation arises from the evolving nature of AI in cardiology, which lacks clearly defined conceptual boundaries. Consequently, using the search term “AI,” we retrieved documents related to AI in other medical fields. Despite this limitation, our bibliometric review has effectively outlined the current state of AI in cardiology. Therefore, our findings serve as a foundational reference point for future reviews tracking the evolution of this field.

Additionally, this study is limited to journal publications. Future research will expand the scope of this topic to include books, dissertations, and conference papers. Furthermore, while only journal papers in English were considered, there is potential value in analyzing publications authored in languages such as French, Croatian, Portuguese, and Spanish. Furthermore, the exclusion of articles outside the time range of 2013-2024 and those in other areas of medicine besides the cardiovascular system, such as physics, chemistry, environmental, material sciences, art and humanities, veterinary, planetary sciences, and psychology, might have greatly affected the outcome of this study. Limiting this research to articles’ abstracts and titles may also have a huge impact on the results.

## Conclusions

This bibliometric analysis of 1,719 articles published from January 2013 to June 2024 provides a comprehensive overview of the current state of research on the application of AI in cardiology. Our study highlights the rapid growth and increasing significance of this interdisciplinary field, marked by a substantial rise in publications, particularly over the past decade. The United States, China, and several European countries are leading contributors, reflecting their strong research infrastructures and commitment to advancing healthcare through technological innovation. Key institutions, including Stanford University, Havard Medical School, Mayo Clinic, and the University of Oxford, and influential authors like Deo RC (United States), Slomka PJ (United States), and Reuckert D (United Kingdom), have been identified, showing the pivotal role played by certain researchers and organizations in driving the field forward. The collaboration networks show a high degree of national and international cooperation, which is important for the development and dissemination of AI technologies in medicine. The co-authorship map identified Dey D (United States), Slomka PJ (United States), and Berman DS (United States) as the most influential co-authors. The studies they performed together on the diagnosis of coronary artery disease have greatly impacted research into AI and ML in cardiology. Slomka PJ, Miller E., and Gransar H are identified as the most prominent co-cited authors. They have published many papers on the role of AI in predicting cardiovascular morbidity and mortality using cardiac diagnostic results.

However, the analysis also identifies gaps and areas for future research. Despite significant progress, there is a need for more studies on the implementation of AI in clinical practice, ethical considerations, and the integration of AI tools into existing healthcare systems. Moreover, research should continue to explore novel AI applications and validate their effectiveness in diverse patient populations. This bibliometric analysis underscores the transformative potential of AI in cardiovascular medicine, highlighting key contributions and emerging trends. It provides valuable insights for researchers, clinicians, and policymakers aiming to harness AI to improve cardiovascular health outcomes. Continued innovation, interdisciplinary collaboration, and a focus on practical implementation will be essential to fully achieve the benefits of AI in this critical field.
